# Update notifications for the BioCyc collection of databases

**DOI:** 10.1093/database/bax086

**Published:** 2017-11-14

**Authors:** Suzanne Paley, Peter D Karp

**Affiliations:** 1 Bioinformatics Research Group, SRI International, 333 Ravenswood Avenue, Menlo Park, CA 94025, USA

## Abstract

We describe the BioCyc update notifications service, a new mechanism to keep researchers informed of the latest developments in their areas of interest. BioCyc.org combines databases for 9,300 sequenced organisms that integrate genome, metabolic pathway, and regulatory information with extensive bioinformatics tools. Users of the BioCyc website can register their specific areas of interest online by specifying a set of genes, pathways and/or Gene Ontology terms. Then, when significant new information becomes available in a BioCyc database in a user’s interest areas (usually due to curation), an email notification is sent to the user. The BioCyc ontology is leveraged to identify changes that are both relevant to a user’s specified interests and worthy of notification. Every effort is made to ensure that the resulting email text is both concise and informative, with links to relevant BioCyc pages.

**Database URL:** https://BioCyc.org, https://EcoCyc.org, https://MetaCyc.org

## Introduction

The BioCyc collection of databases (1) forms a unique and valuable repository of biological knowledge. Particularly for our gold standard databases, EcoCyc (2) and MetaCyc (1), but also for an increasing number of additional organism databases, expert curators devote many hours to compiling information from the biological literature and authoring mini-reviews describing the state of knowledge about genes and pathways. For example, EcoCyc now contains 2900 textbook equivalent pages of mini-reviews derived from 33,000 publications. Every effort is made to keep these resources up-to-date with respect to the latest scientific discoveries. However, until recently, no way existed to close the loop and inform researchers of curation updates relevant to their research interests. Here we describe a new mechanism—the BioCyc update notifications service, by which BioCyc users can register their specific areas of interest online and then receive timely and targeted notifications when new information in their interest areas becomes available in BioCyc.

Updated versions of the BioCyc website and component databases are released three times per year. At those times, a general announcement is sent by email to all registered users of the site. Although this announcement typically outlines significant areas of new or updated curation, the description is by necessity general and incomplete. If a researcher wanted to know if new curation was available for specific entities of interest (e.g. genes), until recently they would have no recourse but to visit the web pages for each of those objects and try to manually discern what information, if any, is new.

### Related work

Other curated model organism databases (MODs) face similar issues. They typically issue news announcements when notable or significant data updates are available, but these announcements are not targeted to individual user interests. MODs that use the InterMine platform (3) enable users to save searches and lists of objects of interest to facilitate regular checks, but they still rely on the user returning to re-execute searches and to check for new information—users are not notified when changes are available nor are they informed as to what in their lists or query results has changed recently. PubMed offers an alert service whereby users can receive email notifications at regular intervals listing new journal articles in their areas of interest as defined by Boolean combinations of keywords. PubMed is not a curated MOD, but its alert service is one model of how researchers can be kept abreast of the latest developments in their field. Motivating us was our hope that the extensive ontologies available within BioCyc would enable us to provide a more precise alerting service for updates to BioCyc.

## Results

Our solution to the alerting problem is enabling the registered users of the BioCyc, EcoCyc and MetaCyc websites to subscribe to specific areas of interest in a given organism database. Then, shortly after every new release, newly curated data that matches a user’s interests results in an email that is sent informing the user of the relevant changes. However, these days, users are deluged with email on a daily basis, and a notification email is not useful unless the user actually reads it. The main challenge we faced was how to tailor the email notifications to ensure that the user receives all relevant information without being inundated by a mass of less relevant details. This challenge breaks down roughly into three components: (i) deciding on an appropriate level of granularity at which to specify areas of interest, and making it simple and convenient for users to register for and manage their notifications; (ii) determining what types of database updates are significant enough to warrant notification; and (iii) generating email content that is sufficiently informative yet not overwhelming to the recipient.

### Registering for and managing notifications

Users can define their interest areas by registering for notifications about specific genes or pathways, about Gene Ontology (GO) terms, and about pathway classes from the MetaCyc pathway ontology (which is present in all BioCyc databases).

Each designation of a gene or a pathway actually encompasses a whole collection of functionally related database objects. When a user registers for a specific gene such as *trpA*, in order to decide whether changes have been made to that gene, the notification software must check not just the gene object but also the gene product; complexes the gene product may belong to; enzymatic or regulatory activities of the gene product or complexes; pathways containing the product or complexes; and transcriptional, translational and substrate-level regulation. When a user registers for a specific pathway, we consider not just the pathway object but also its reactions, metabolites, enzymes, genes and regulatory interactions.

GO terms and pathway classes define sets of genes and pathways, respectively, and can be quite useful for specifying biological interest areas of appropriate specificity. When users subscribe to a GO term such as GO:0001906 (cell killing) or GO:0022610 (biological adhesion), they have two choices: (i) they can choose to be notified only when new genes are annotated to that term, or (ii) they can be notified of changes (using the above criteria for changes associated with a gene) to all genes currently annotated to that term. An analogous choice applies when a user registers for a pathway class such as secondary metabolites biosynthesis, chlorinated compounds degradation, or arsenate detoxification. All object designations are specific to a single database (e.g. a user who registers interest in a pathway in BsubCyc, the database for *Bacillus subtilis*, will only be notified of changes to the pathway and its enzymes in that database, not to the corresponding pathway in EcoCyc, MetaCyc or any other organism).

From every page for a gene, pathway, GO term, or pathway class on the BioCyc website, the right-sidebar Operations menu includes a link to subscribe to notifications for that object. Users may also access a dedicated page (Analysis->Update Notifications, accessible from every BioCyc page) where they can either enter (with autocomplete) the objects they wish to subscribe to or cancel any object notifications they are no longer interested in. If users have a large list of specific genes or pathways that they wish to subscribe to (for example, all genes that form the result of a particular query, or that are associated with some experiment), they can create a SmartTable (4) containing all desired objects and subscribe to everything in the SmartTable with a single command. Thus, we make it easy for users to register their interests as broadly or narrowly as they choose.

### Determination of significant changes

From one release to the next, a database object can undergo changes for a variety of reasons, and to any number of different fields. We do not want to trigger update notifications for all of these changes. A curator may update an enzyme to correct a typo or erroneous data value, to add a synonym or minor clarification, or to reflect data from a newly curated publication. All of these would be considered curated changes, yet it is our judgment that most users would prefer to be notified only of the latter, but not of any of the former. Databases also undergo periodic programmatic updates. Often these updates represent only minor changes in schema or representation that are unworthy of notification; but sometimes, they involve integration of new data sources that are likely to be of significant interest. Other types of changes that we consider unworthy of notification (whether done by curator or programmatically) include when redundant objects are merged, obsolete objects or GO annotations are deleted, and various fixes are applied to maintain database consistency, even though all these operations are likely to involve multiple changes to existing database objects.

Although we can programmatically compare any object in a new DB release with the same object in the previous version and determine what changes have been made, determining from that comparison the reason for each change, and whether or not it reflects a significant change about which users should be notified, is not straightforward.

Fortunately, we can leverage the fact that BioCyc curators never make significant changes to an object without adding a relevant citation (either to the changed attribute itself or to the object as a whole). Similarly, the importation of a significant source of new data can also be expected to be accompanied by a new citation. Thus, regardless of the number and type of changes, a gene or pathway is considered to have undergone significant updates that warrant notification only if at least one new citation (not present in the previous release) is associated with it or any of its associated objects or attributes. Once we have determined via this filter that a particular object meets the threshold for notification, we collect all differences between the old and new versions (since we often cannot know which specific field changes are associated with the new citations) to be summarized in the notification email.

### Generation of email contents

**Figure 1.**
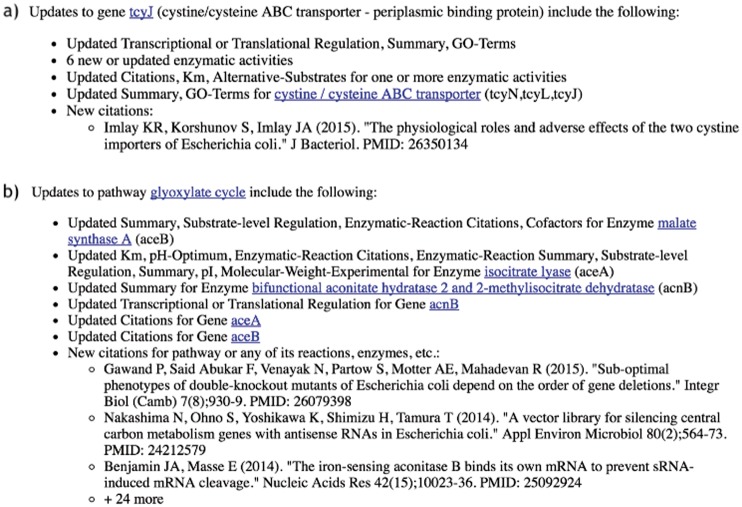
**(a)** Sample notification text for an updated gene. **(b)** Sample notification text for an updated pathway. Underlined text are hyperlinks to BioCyc pages.

The purpose of the notification email is not to reproduce the contents of any BioCyc web page. Rather, as compactly and concisely as possible, we want to provide users with links to updated pages in their areas of interest, and enough information about the types of changes that they can both decide if clicking any given link is worthwhile, and, if so, where on the page they should look for the changes. For example, if a user is subscribed to a pathway, and there are changes to two enzymes in the pathway, the email would contain a link to the pathway page and then list the changed enzymes with links to those enzyme pages. For each changed enzyme, a listing of the changes, such as changes to transcriptional or translational regulation, changes to the textual summary, GO terms, etc. would be given. Finally, we include the list of new publications. Figure 1a and b show sample text generated for a gene and a pathway respectively, and are representative of the level of detail that users can expect in the email message. We believe this level of reporting will enable users to quickly determine whether or not the changes are of interest to them, and, if not, to rapidly scroll past. If a user is subscribed to multiple changed objects, all notifications for a single organism or database are combined into a single email message. The email message body includes a prominent link to the page to manage or unsubscribe from notifications. Because BioCyc releases occur only three times per year, with at most one email per release, these messages should not contribute appreciably to inbox clutter.

## Conclusions

We have developed a software tool for notifying users of significant updates to curated BioCyc databases. Rather than specifying interest areas using keywords, as with previous notification systems, interest areas are specified using ontologies (Gene Ontology or the MetaCyc pathway ontology) and database objects to provide much higher precision. Notification emails are triggered when a new citation is added to an object within a user’s defined interest area. Notification emails are both comprehensive and concise to appeal to busy biologists. It is our hope that many BioCyc users will avail themselves of this opportunity to stay up-to-date with the latest developments in their fields of interest.
